# Genetic evidence reveals a causal relationship between rheumatoid arthritis and interstitial lung disease

**DOI:** 10.3389/fgene.2024.1395315

**Published:** 2024-05-14

**Authors:** Rong Zhao, Yi-Wen Zhang, Jin-Cheng Guo, Jun Qiao, Shan Song, Ting-Ting Zhang, He-Yi Zhang, Sheng-Xiao Zhang

**Affiliations:** ^1^ Department of Rheumatology, Second Hospital of Shanxi Medical University, Taiyuan, Shanxi, China; ^2^ Key Laboratory of Cellular Physiology at Shanxi Medical University, Ministry of Education, Taiyuan, China

**Keywords:** rheumatoid arthritis, interstitial lung disease, Mendelian randomization, causal relationship, gene

## Abstract

**Background/purpose:** Previous epidemiological studies have associated interstitial lung disease (ILD) with rheumatoid arthritis (RA), yet the causality of this relationship remains uncertain. This study aimed to investigate the genetic causal link between ILD and RA.

**Methods:** Genome-wide association study (GWAS) statistics for ILD and RA were collected from public datasets. Relevant single-nucleotide polymorphisms (SNPs) were selected by executing quality control steps from the GWAS summary results. A two-sample bidirectional Mendelian randomization (MR) analysis was performed to assess the causal relationship between the two conditions. The MR analysis primarily used the inverse variance weighting (IVW), weighted median (WM), and MR-Egger regression methods. Sensitivity analyses, including MR-Egger, leave-one-out, and MR Pleiotropy RESidual Sum and Outlier (MR-PRESSO), were conducted to evaluate the heterogeneity and pleiotropy. Replication analyses using Asian datasets were also conducted to enhance the robustness of our findings.

**Results:** In the European population, RA was found to increase the risk of ILD by 9.6% (OR: 1.096, 95% CI: 1.023–1.174, *p* = 0.009). Conversely, ILD was associated with a 12.8% increased risk of RA (OR: 1.128, 95% CI: 1.013–1.256, *p* = 0.029). Replication analyses from Asian GWAS further supported these findings, particularly the increased risk of ILD attributable to RA (OR: 1.33, 95% CI: 1.18–1.49, *p*-value <0.001).

**Conclusion:** Our findings underscore the clinical importance of screening for ILD in RA patients and suggest that effective management of RA could significantly benefit ILD patients. The potential applicability of novel RA treatments to ILD warrants further exploration. Additionally, racial disparities in the manifestation of these diseases should not be overlooked, as they may offer new perspectives for targeted therapies in diverse populations.

## 1 Introduction

Rheumatoid arthritis (RA) is a chronic, systemic autoimmune disease whose main characteristic is persistent joint inflammation that can lead to lifelong damage and loss of function (Cojocaru et al., 2010). It affects approximately 1% of the world’s population and 0.32%–0.36% in China (Chen et al., 2015). The exact etiology of RA is unknown, but it has been established that its chronic course could be correlated with genetic factors, and the heritability of RA was estimated to be 60% (Yarwood et al., 2016). So far, using genome-wide association studies (GWAS), research studies have revealed that several genetic susceptibility loci play a vital role in RA, such as the human leukocyte antigen D-related B1 gene (*HLA-DRB1*) and protein tyrosine phosphatase non-receptor type 22 (*PTPN22*) (Tanaka, 2020).

Interstitial lung disease (ILD) is a large heterogeneous group of diseases affecting the lung parenchyma through inflammation and fibrosis, characterized by symptoms of cough, shortness of breath, and hypoxia (Newton et al., 2016; Rivera-Ortega and Molina-Molina, 2019). Epidemiological surveys estimate the prevalence of IDL to be as high as 24.9% (Fazeli et al., 2021). Genetic factors are considered important risk factors for ILD (García-Sancho et al., 2011; Rivera-Ortega and Molina-Molina, 2019). Currently, many alleles strongly associated with ILD risk have been identified by GWAS, such as *MUC5B* (Newton et al., 2018), *HLA-DRB1* (McDermott et al., 2021), and the gene encoding surface active protein C (*SFTPC*) (Kropski, 2020).

The association between RA and ILD has been extensively discussed. In some studies, ILD was identified as a severe extra-articular manifestation of RA (Esposito et al., 2019; England and Hershberger, 2020). A prospective registry study noted that patients with a combination of rheumatoid factor (RF) or anti-citrullinated protein antibody (ACPA) seropositivity had a higher prevalence of ILD when compared to seronegative subjects (Natalini et al., 2021; Holers et al., 2022). In addition, studies have also shown that significant lung inflammation may be associated with higher local and systemic ACPA concentrations (Natalini et al., 2021). Gregory C McDermott et al. reported that a substantial proportion of patients usually develop ILD prior to the manifestation of arthritis, suggesting a role for the lungs in the progression of RA disease (McDermott et al., 2021). Similarly, studies have shown that 10%–17% of patients were diagnosed with ILD before they were diagnosed with RA (Hyldgaard et al., 2017). However, the causal relationship between the two conditions still needs to be fully understood. Therefore, it is of great interest to establish their association with more basic evidence and elucidate the mechanisms underlying the association between RA and ILD.

Mendelian randomization (MR) is an epidemiological method that can infer causality between the exposure and outcome by using single-nucleotide polymorphisms (SNPs) as an instrumental variable (IV) (Burgess et al., 2013). This method is not prone to reverse causation since disease states usually do not change the germline DNA sequences. Moreover, MR can limit confounding since genotypes are randomly assorted at meiosis (Manousaki et al., 2021). Though well-designed randomized controlled trials (RCTs) are usually the best approach to estimating a causal relationship between a risk factor and a disease, their implementation is limited by the small sample size, limited external validity, short duration of an intervention, and ethical concerns that limit the implementation of RCTs. Thus, MR has specific advantages that can complement another study (Li et al., 2017). This study took advantage of MR and investigated the causal relationship between RA and ILD using genetic determinants estimated from GWAS summary statistics of different populations.

## 2 Materials and methods

### 2.1 Study design

**FIGURE 1:**
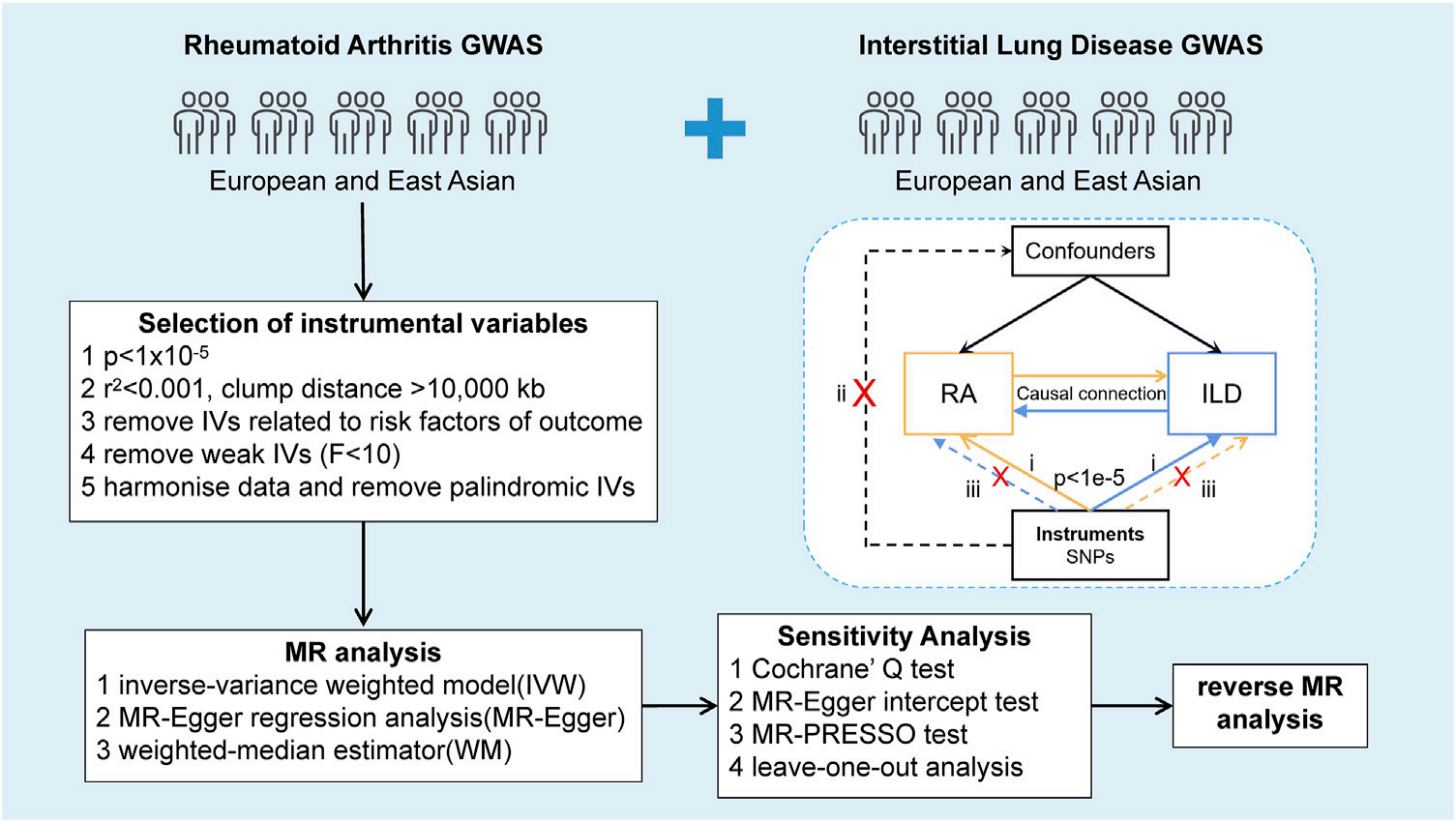
Study design of the bidirectional Mendelian randomization between RA and ILD. The forward MR analysis evaluating the causal effects of RA on ILD was indicated with yellow solid line arrows. In contrast, the reverse MR analysis assessing the causal impact of ILD on RA was indicated with blue solid line arrows. Dashed lines represented irrelevant links, while the cross showed an impassable association between SNPs and confounders or the outcome.

MR analysis was performed to investigate the causal relationship between RA and ILD. Relevant SNPs were selected as IVs by executing quality control steps from the GWAS summary results, which need to meet three basic assumptions (Figure 1): (i) SNPs were strongly associated with exposure (correlation hypothesis); (ii) SNPs were not associated with confounding factors, meaning that the results were not affected by confounding factors (independence assumption); and (iii) SNPs were not associated with the outcome or did not directly affect the outcome (exclusivity hypothesis). Based on the previous research, we have listed the STROBE-MR checklist of MR studies to ensure the integrity of our processes, as shown in Supplementary Table S1 (Skrivankova et al., 2021a; Skrivankova et al., 2021b).

### 2.2 Data sources

#### 2.2.1 Genetic datasets associated with rheumatoid arthritis

The RA-related outcome dataset of Europeans, derived from a summary of the GWAS previously communicated by Okada et al., included 14,361 cases and 43,923 healthy controls of European descent, totaling 8,747,963 RA-related SNPs (Okada et al., 2014). Genetic datasets associated with RA in the East Asian population are derived from an OpenGWAS project previously compiled by Sakaue Scott et al. (2013), containing 1,046 cases and 176,974 healthy controls, and the number of SNPs is 12,454,608. All RA cases met the 1987 RA diagnostic criteria of the American College of Rheumatology (Arnett et al., 1988) or were diagnosed with RA by a specialist rheumatologist.

#### 2.2.2 Genetic datasets for interstitial lung disease

Genetic prediction of the ILD-related gene GWAS data was obtained from the available meta-analysis of published GWASs. The European dataset contains 1,969 cases and 196,986 healthy controls, covering 16,380,417 genotyped SNPs. Genetic datasets for ILD contain 806 cases and 211,647 healthy controls from Ishigaki K et al., covering 8,885,805 SNPs from East Asia (Sakaue et al., 2021). All cases were diagnosed according to the American Thoracic Society and the European Respiratory Society guidelines.

Since this MR study was conducted using publicly available GWAS summary data, ethical approval and informed consent given all subjects could be found in the original publications.

### 2.3 Selection of instrumental variables

Strict filtering was performed to control SNP quality before MR analysis. First, we extracted SNPs associated with the genome-wide significance threshold of exposure (*p* < 1 × 10^−5^). Second, for the condition of independence from exposure, we selected SNPs with independent inheritance (*r* ^2^ < 0.001) and no linkage disequilibrium (LD, clump distance >10,000 kb) in the summary statistics. Then, to avoid possible statistical bias from the original GWAS, we excluded IVs with minor allele frequency (MAF) less than the threshold of 0.01. Finally, the strongly correlated IVs were obtained by the F statistic >10 (Burgess et al., 2011; Pierce and Burgess, 2013).

Furthermore, it is confirmed that BMI, smoking, and alcohol consumption were implemented with a varying degree of effects on RA and ILD in previous studies (Baka et al., 2009; Maxwell et al., 2010; Chang et al., 2014; George and Baker, 2016; Poudel et al., 2020). Based on this, the PhenoScanner (http://phenoscanner.medschl.cam.ac.uk) search was applied to remove these variants, which are associated with confounding factors. At the same time, the SNPs selected from the exposure and outcome datasets were harmonized, removing the palindromic SNPs using intermediate allele frequencies. Finally, the remaining IVs were used as tools for further MR analysis.

### 2.4 Mendelian randomization analyses

The preliminary analysis of this study used inverse variance weighting (IVW), weighted median (WM), and MR-Egger regression to estimate the causal relationship between the exposure and outcome. The IVW approach calculated the Wald ratio for each SNP on the outcome and obtained a pooled causal estimate, and this method allows for overdispersion. WM estimates were used to complement the IVW approach for reliable estimation and accounted for at least 50% of the analysis weight provided by the validation IV. The MR-Egger method was used to test for bias in the results of analyses that result from influencing the results through pathways other than exposure. Of these methods, IVW was the most crucial method for estimating causality between the exposure and outcome, which provides an accurate estimate of the causal effect of the outcome risk. These MR methods are detailed in published studies (Didelez and Sheehan, 2007; Davey Smith and Hemani, 2014).

### 2.5 Sensitivity analysis

To assess the robustness of these results and prevent potential pleiotropy and heterogeneity, a series of sensitivity analyses were conducted. Cochran’s Q test was applied to quantify the heterogeneity between SNPs and was visualized with funnel plots. The intercept term of MR-Egger regression and the MR-PRESSO global test was used to assess whether horizontal pleiotropy affected the results of MR analysis (Egger et al., 1997). In addition, leave-one-out analysis was performed to determine whether any single SNP drove the causal estimates. Results were examined as statistically significant at *p* < 0.05. All analyses mentioned above were implemented in R V4.1.0 using the “TwoSampleMR” package (Hemani et al., 2018).

## 3 Results

### 3.1 Causal effects of RA on ILD

We incorporated 104 SNPs with a *p*-value less than 1 × 10^−5^ as IV SNPs. All SNPs were consistent with the F statistic >10. Twelve SNPs related to RA were removed for being palindromic with intermediate allele frequencies, and the potential confounding factors found in the PhenoScanner including rs10774624 (associated with smoking), rs34695944 (associated with alcohol consumption), and rs4272, rs34431565, rs773125, rs10857135, rs2736337, and rs9275183 (associated with body mass index, BMI) were removed. Then, the remaining 84 SNPs were used as IVs for subsequent MR analysis. Detailed information of IVs for RA is presented in Supplementary Table S2.

**FIGURE 2:**
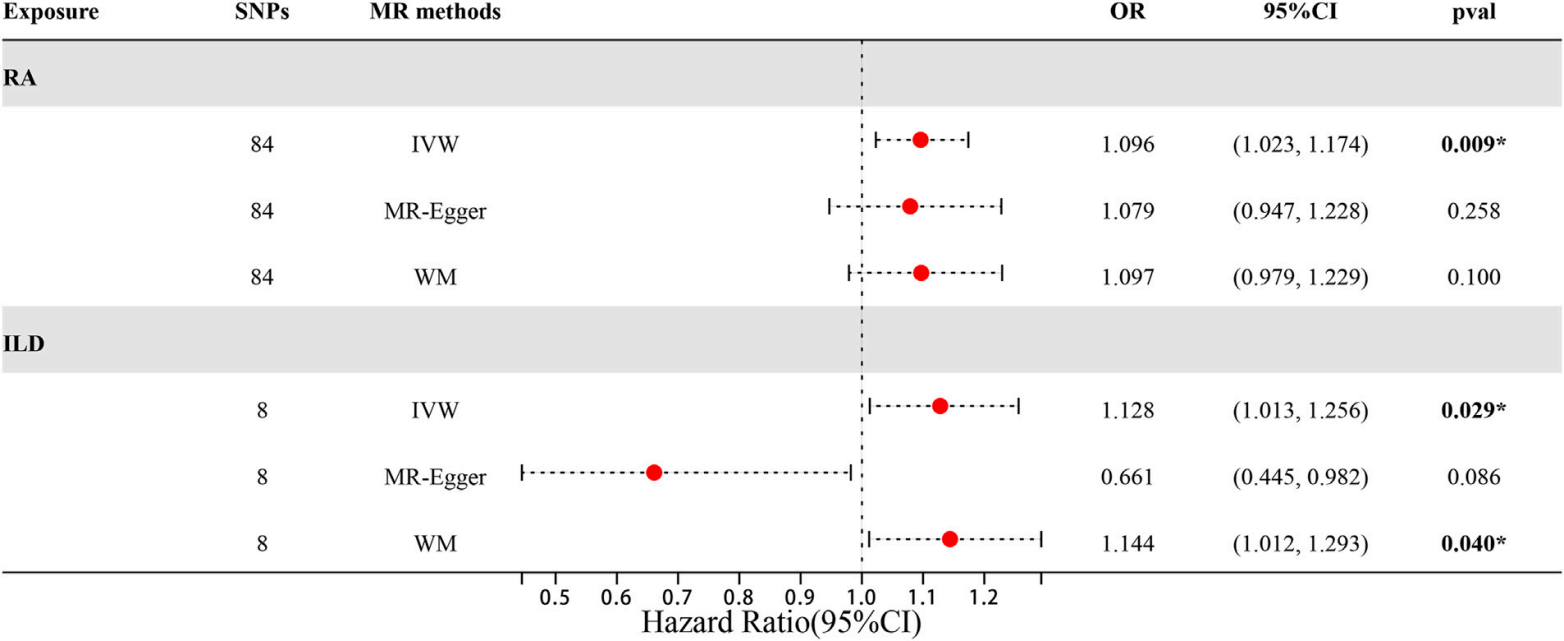
Forest plots of MR results by IVW, MR-Egger, and weighted median in the group of European population.

**FIGURE 3:**
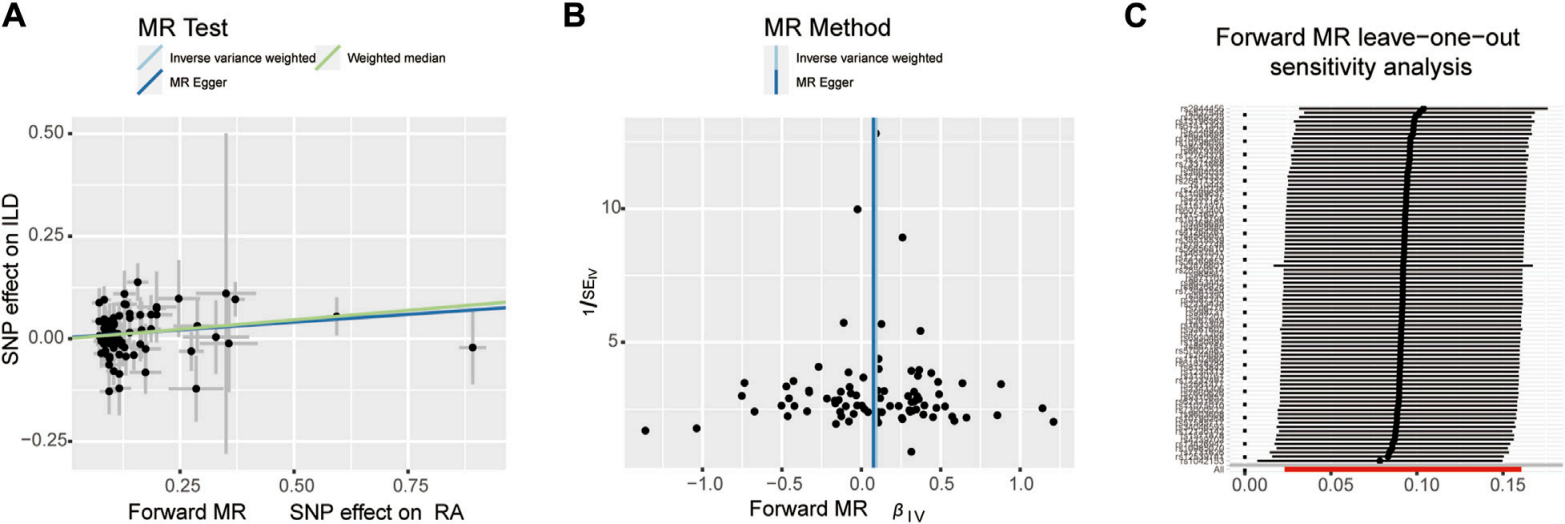
Scatter plots, funnel plots, and leave-one-out analysis were used for forward MR analysis in the group of European population. **(A)** Scatter plots for two-sample Mendelian randomization analysis of the effect of RA on ILD using the IVW, in conjunction with MR-Egger and WM methods. **(B)** Funnel plots of the SNPs enrolled in the causality estimates of RA effects on genetically predicted ILD. The funnel plot displayed a symmetric pattern of effect size variation around the point estimate. IVW and MR-Egger regression slopes were used to explore asymmetry as a sign of pleiotropy. **(C)** Leave-one-out analysis was performed to evaluate whether every single IV drove the causal association of RA on ILD disproportionately.

When setting ILD as the outcome, RA was causally associated with ILD, as shown in Table 1 and Figures 2, 3A. According to the IVW analysis, the presence of RA may increase 9.6% risk of ILD (OR: 1.096, 95% CI: 1.023–1.174, *p* = 0.009). Sensitivity analysis shows that there was heterogeneity among the IVs (Q = 109.17, PQ = 0.029, Figure 3B), while the MR-Egger intercept analysis suggested no evidence of horizontal pleiotropy (*p* = 0.772). Furthermore, leave-one-out plots suggested that the causal estimates were unlikely to be influenced by certain SNPs. This suggested that the findings were stable and reliable (Figure 3C).

**TABLE 1:** Causal effects of RA on ILD in European population.

Exposure	Outcome	SNP	MR method	OR	95% CI	*p*-val	Q_*p*-val
RA	ILD	84	IVW	1.096	(1.023, 1.174)	0.009*	0.029*
			MR-Egger	1.079	(0.947, 1.228)	0.258	0.025*
			WM	1.097	(0.979, 1.229)	0.100	-
ILD	RA	8	IVW	1.128	(1.013, 1.256)	0.029*	0.170
			MR-Egger	0.661	(0.445, 0.982)	0.086	0.812
			WM	1.144	(1.012, 1.293)	0.040*	-

Odds ratio (OR) values, confidence interval (CI), *p*-val and Q_*p-*val of MR **r**esults were obtained by IVW, MR-Egger, and weighted median in the group of European population. *p < 0.05.

### 3.2 Causal effects of ILD on RA

There were 25 SNPs that were significantly associated with ILD (*p* < 1 × 10^−5^) and were incorporated as IVs. Similarly, the F-statistic values of these IVs were all more than 10. The palindromic alleles were then removed and synergized with RA-associated SNPs, and the remaining eight SNPs were used as IVs for subsequent MR analysis. Detailed information of IVs for ILD is presented in Supplementary Table S3.

**FIGURE 4:**
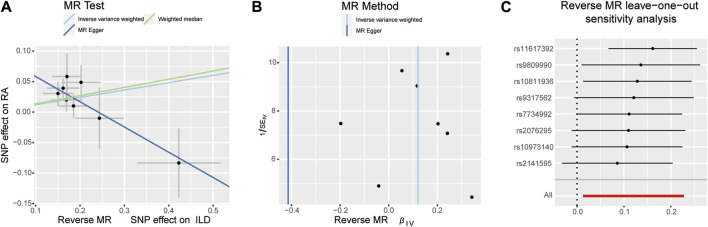
Scatter plots, funnel plots, and leave-one-out analysis were conducted for the reverse MR analysis in the group of European population. **(A)** Scatter plots for IVW, MR-Egger, and WM analysis methods highlighted the effect of ILD on RA. **(B)** Funnel plots of ILD genetic liability effects on RA. IVW and MR-Egger regression slopes were used to explore asymmetry as a sign of pleiotropy, with the vertical line in the middle indicating the sum of different effect sizes. **(C)** Leave-one-out analysis was used to determine whether any single SNP drove the causal association of ILD on RA, which repeated the IVW analysis by discarding each exposure-related SNP.

When setting RA as the outcome, ILD was also causally associated with RA (Table 1; Figures 2, 4A). The existence of ILD may increase the risk of RA by 12.8% (OR: 1.128, 95% CI: 1.013–1.256, PQ = 0.029). Results of the weighted median method also support our findings (OR: 1.144, 95% CI: 1.012–1.293, *p* = 0.04). In the analysis of sensitivity, Cochran’s Q test (Q = 2.97, *p* = 0.812, Figure 4B) indicated no heterogeneity in the causal effect between ILD and RA. No significant directional horizontal pleiotropy between RA and ILD was presented in the MR-Egger regression analysis. In the leave-one-out sensitivity analysis, no single SNP significantly biased the causal effect of ILD on RA (Figure 4C).

### 3.3 Replication analyses in East Asian population

#### 3.3.1 Causal effects of RA on ILD

There were 46 SNPs that were significantly associated with ILD without linkage disequilibrium (*r* ^2^ < 0.001, *p* < 1 × 10^−5^). The confounding factors such as rs10821944, rs111335405, rs2069235, rs2647192, rs4728142, rs7732397, and rs2618476 (all associated with the body mass index) found in the PhenoScanner were removed. Then, the palindromic alleles were removed and synergized with ILD-associated SNPs. The remaining 36 SNPs were used as IVs for subsequent MR analysis. Details of IVs are presented in Supplementary Table S4.

**FIGURE 5:**
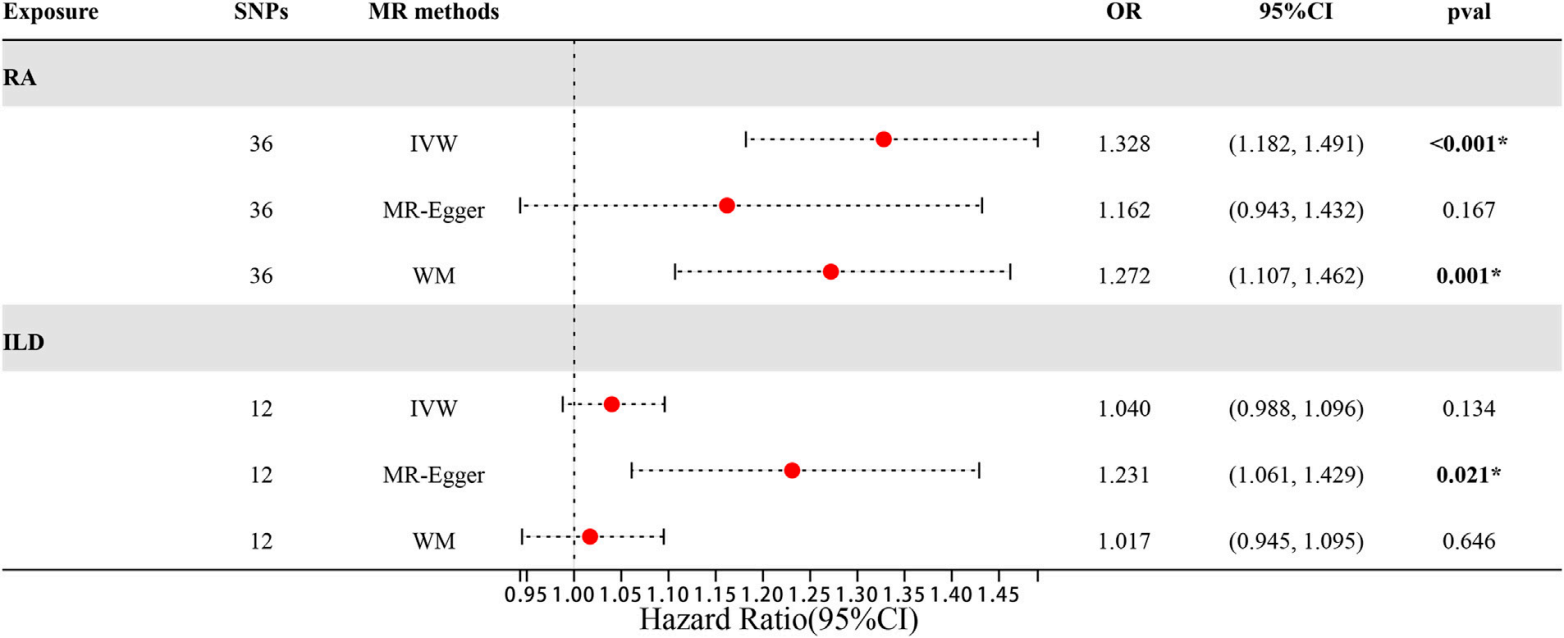
Forest plots of MR results by IVW, MR-Egger, and weighted median in the group of East Asian population.

**FIGURE 6:**
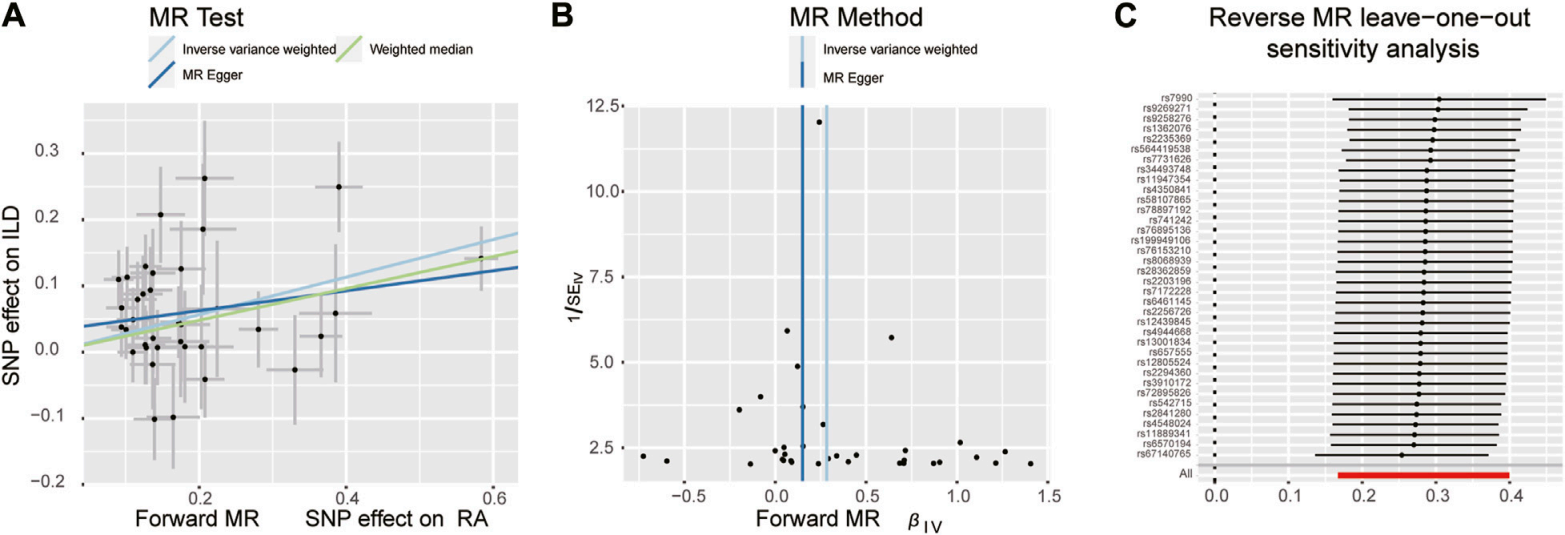
Scatter plots, funnel plots, and leave-one-out analysis were conducted for forward MR analysis in the group of East Asian population. **(A)** Scatter plots for two-sample Mendelian randomization analysis of the effect of RA on ILD using the IVW, in conjunction with MR-Egger and WM methods. **(B)** Funnel plot of the SNPs enrolled in the causality estimates of RA effects on genetically predicted ILD. The funnel plot displayed a symmetric pattern of effect size variation around the point estimate. IVW and MR-Egger regression slopes were used to explore asymmetry as a sign of pleiotropy. **(C)** Leave-one-out analysis was conducted to evaluate whether every single IV drove the causal association of RA on ILD disproportionately.

The MR estimates of different methods are presented in Table 2 and Figures 5, 6A. Notably, the results showed a strong causal relationship between RA and ILD (OR: 1.33, 95% CI: 1.18–1.49, *p* < 0.001). The result of weighted median methods further endorsed the causal effect (OR: 1.27, 95% CI: 1.11–1.46, *p* < 0.001). As for sensitivity analysis, we found that there was heterogeneity among the IVs (Q = 49.27, PQ = 0.043, Figure 6B). Furthermore, the MR-Egger intercept analysis shows that there was no evidence of horizontal pleiotropy (*p* = 0.144). A leave-one-out analysis was conducted to avoid horizontal pleiotropy caused by a single SNP (Figure 6C).

**TABLE 2:** Causal effects of RA on ILD in East Asian population.

Exposure	Outcome	SNP	MR method	OR	95% CI	*p-*val	Q_*p*-val
RA	ILD	36	IVW	1.328	(1.182, 1.491)	<0.001*	0.029*
			MR-Egger	1.162	(0.943, 1.432)	0.167	0.044*
			WM	1.272	(1.107, 1.462)	0.001*	-
ILD	RA	12	IVW	1.040	(0.988, 1.096)	0.134	0.457
			MR-Egger	1.231	(1.061, 1.429)	0.021*	0.873
			WM	1.017	(0.945, 1.095)	0.646	-

Odds ratio (OR) values, confidence interval (CI), *p*-val and Q_*p-*val of MR results were obtained by IVW, MR-Egger, and weighted median in the group of East Asian population. *p < 0.05.

#### 3.3.2 Causal effects of ILD on RA

**FIGURE 7:**
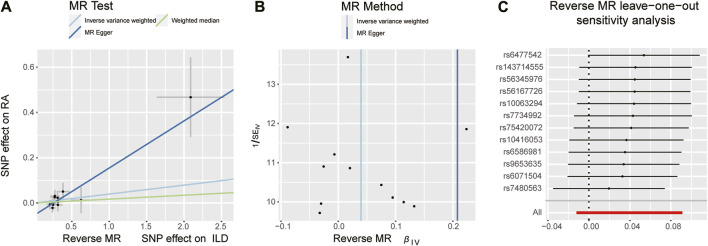
Scatter plots, funnel plots, and leave-one-out analysis were conducted for the reverse MR analysis in the group of East Asian population. **(A)** Scatter plots for IVW, MR-Egger, and WM analysis methods highlighted the effect of ILD on RA. **(B)** Funnel plot of ILD genetic liability effects on RA. IVW and MR-Egger regression slopes were used to explore asymmetry as a sign of pleiotropy, with the vertical line in the middle indicating the sum of different effect sizes. **(C)** Leave-one-out analysis was used to determine whether any single SNP drove the causal association of ILD on RA, which repeated the IVW analysis by discarding each exposure-related SNP.

There were 14 SNPs that were significantly associated with ILD without linkage disequilibrium (*r* ^2^ < 0.001, *p* < 1 × 10^−5^). The confounder rs3129960 (associated with the body mass index) found in the PhenoScanner was removed. Then, the remaining 12 SNPs were used as IVs for subsequent MR analysis. Details of IVs are presented in Supplementary Table S5. Horizontal pleiotropy was unlikely to skew the causality of ILD with RA, according to the results of the MR-PRESSO global test (*p* = 0.542). However, results of IVW showed that an increase in the risk of having ILD was not statistically related to an increased risk of having RA (RA: OR = 1.040, 95% CI: 0.997–1.10, *p* = 0.13). The detailed information is presented in Table 2 and Figures 5, 7A). Furthermore, no significant evidence of horizontal pleiotropy was observed for IVs (Q = 5.27, PQ = 0.873) (Figure 7B), and leave-one-out plots suggested that the causal estimates were unlikely to be influenced by a single SNP (Figure 7C).

## 4 Discussion

Our study suggested that the inflammatory process of RA had a positive causal effect on the presence of ILD and *vice versa* in the European population. However, in the Asian population, we only found that the presence of RA caused an increased risk of ILD. This is the first study to explore the bidirectional causal relationship between RA and ILD through a two-sample MR approach based on GWAS summary statistics, providing evidence of a genetic link between RA and ILD. Our findings indicated that these two common autoimmune diseases may share similar underlying pathophysiological mechanisms, but racial differences cannot be ignored.

Many studies have found that RA increases the risk of developing ILD. For example, in a longitudinal study, 51% of patients were diagnosed with RA–ILD more than 5 years after diagnosing RA (Mohning et al., 2021). In an incident cohort of RA patients in the UK, 4% developed clinically significant RA–ILD on high-resolution CT (HR-CT) imaging during 15 years of follow-up (Duarte et al., 2019). In a smaller study of RA–ILD patients in China, ILD was diagnosed after RA in 69% of cases, and the median time between RA and RA–ILD diagnosis was 60 months (Chen et al., 2021). However, not every study has reached the same conclusion about the association between RA and ILD. In contrast, some studies have concluded that the lungs may be a mucosal (and potentially an initiating) site for generating RA-related autoimmunity (Gizinski et al., 2009; Demoruelle et al., 2012). A study predominantly about middle-aged women of European ancestry showed that pulmonary involvement was present early in the disease course in RA (Wilsher et al., 2012). Moreover, in a survey conducted on 10%–20% of RA cases, respiratory symptoms may precede the onset of articular symptoms (Kadura and Raghu, 2021). Similarly, studies of RA–ILD patients from Denmark, the United States, and China showed that 10%–17% of patients were diagnosed with ILD before the articular diagnosis of RA (Hyldgaard et al., 2017). Due to their mechanistic complexity and proximity, it can be seen that there is a debate about the cause and the effect of the relationship between RA and ILD. Our bi-directional MR study synthesized the two-sided stance, further complementing previous studies that the two conditions may share similar underlying pathophysiological mechanisms.

There are several possible reasons why RA can increase the risk of developing ILD. Ayodeji Adegunsoye et al. generalized that under the regulation of some harmful environmental factors such as smoking, diet, viral infection, and ambient air pollutants, individuals with genetic variation are more susceptible to pulmonary fibrosis (PF) through epigenetic modifications such as DNA methylation, which is referred to as the gene–environment interaction. Among them, the variations of the *MUC5B* promoter are one of the most common genetic risk factors of early and established PF (Adegunsoye et al., 2024). Similarly, a seminal study by Juge et al. in France showed that the *MUC5B* promoter variant is associated with an increased risk of developing ILD in patients with RA (Juge et al., 2018). Building on this finding, a Finnish study, by combining large-scale genotype data with clinical data from a national healthcare registry, found that among RA patients, the lifetime risk of developing ILD was 16.8% for *MUC5B* carriers and 6.1% for *MUC5B* non-carriers (Palomaki et al., 2021). In addition, the role of *MUC5B* SNPs in the developing RA–ILD may be associated with age factor. It significantly increased the risk of RA–ILD early in the RA course (before or within 2 years of RA diagnosis) and after 55 years of age (McDermott et al., 2022). In particular, in a large real-life European multicenter idiopathic pulmonary fibrosis (IPF) study, van der Vis JJ et al. showed that *MUC5B* minor allele carriers were significantly older at diagnosis (*p* = 0.001), and among patients ≥56 years of age at diagnosis, the 3-year cumulative incidence of death was lower among *MUC5B* minor allele carriers (39%) than among non-carriers (57%) (van der Vis et al., 2023).

In addition, several studies suggested that ILD exacerbation was due to the inflammatory process of RA. For example, high titers of rheumatoid factor [RF] and anti-cyclic citrullinated peptide [CCP] antibodies are specifically associated with the development of ILD in patients with RA (Inui et al., 2008; Giles et al., 2014; Kelly et al., 2014; Akiyama and Kaneko, 2022), in which the HLA-DRB1*15 allele may confer a specific susceptibility to the production of anti-citrullinated fibrinogen antibodies (Gyetvai et al., 2010). More specifically, the combination of matrix metalloproteinase-7 (MMP-7), pulmonary and activation-regulated chemokine, and surfactant protein D enhances the above association (Doyle et al., 2015). Among them, MMP-7 was identified as a potential biomarker for RA–ILD and was significantly elevated in the serum of RA patients with clinical and subclinical ILD (Chen et al., 2015). A prospective cohort study evaluated the association of plasma matrix metalloproteinases (MMPs) with the incidence of interstitial lung disease in patients with rheumatoid arthritis in a large multicenter RA cohort, further supporting the potential pathogenic role of MMP-7 and MMP-9 for RA–ILD (Luedders et al., 2024). The association of RA-induced pulmonary fibrosis also involves MHC gene loci on chromosome 6, such as *HLA-B54*, HLA-DQ1B*0601, *HLA-B40*, and sites encoding a-1 protease inhibitors, which are associated with increased ILD risk in patients with RA (Spagnolo et al., 2014).

Although the exact mechanism of how ILD increases the risk of RA is unknown, several possible explanations exist for the results of reverse MR analysis. According to the “mucosal origins hypothesis,” RA-related autoimmunity disorder is initiated at a mucosal site, including the lungs, and then transitions to involve the synovial joints (Holers et al., 2018; Lucchino et al., 2019). In a genetically susceptible individual, injury to the alveoli, airway epithelium, and mucosa caused by smoking, microbial dysbiosis, or other inhalant exposures can lead to citrullination of protein, production of neutrophil extracellular traps, generation of local pulmonary mucosal RA-associated autoantibodies, and establishing of systemic autoimmunity ultimately (Catrina et al., 2014; Valesini et al., 2015; Scher et al., 2016; Friedlander et al., 2020; Prisco et al., 2020). Specifically, in genetically susceptible individuals, the formation rate of the citrullinated protein is high in lung tissues, and citrullination can trigger an immune response leading to the production of ACPA, resulting in an increased risk of RA in patients (Bongartz et al., 2010; Paulin et al., 2015). Another possible explanation is that the elevated cytokines and chemokines caused by the chronic inflammatory state in lung mucosal sites also affect lymphocyte subsets such as T cells, B cells, and macrophages, which recirculate and populate other sites and, in the process, influence the immune status in other tissues participating in the autoimmune response (Rangel-Moreno et al., 2006; Holers et al., 2018; Lucchino et al., 2019). However, findings about ILD increasing the risk of RA were not so consistent in patients with different races in our study. McFarlane et al. mentioned in their study that conflicting reports exist regarding the racial distribution of RA–ILD (McFarlane et al., 2019). Furthermore, disease activity of RA remained significantly different across the Asian groups *versus* European groups in the research of Greenberg et al. (2013). These factors may have a substantial effect on the results, so further studies are needed to identify the genetic differences.

This study has several strengths. First, it is the first bidirectional two-sample MR analysis to reveal a causal relationship between RA and ILD. Second, the screening of instrumental variables set stringent conditions and therefore had high statistical power, allowing for a more robust analysis of possible causal relationships. Finally, in contrast to randomized controlled trials, MR uses an index of genetic variation to measure the causality of disease-related risk factors, overcoming the bias caused by confounding or reverse causality inherent in observational studies.

At the same time, this study has some limitations. First, the study population included in the MR analysis was of European and East Asian origin, and it remains to be verified whether the results represent the whole population. In addition, the minority differences associated with race cannot be ignored in the two diseases, which is worthy of further analysis. Second, though we have taken some steps to exclude heterogeneity and horizontal pleiotropy, the influence on the results is undetermined, and further analysis and studies are still warranted to understand the underlying mechanisms. Finally, it was impossible to determine whether there were overlapping participants in the GWAS data for the exposures and outcomes involved in this study. Fortunately, using strong instruments in this study (e.g., an F statistic much greater than 10) should minimize the potential bias of sample overlap.

In conclusion, our study suggested a causal relationship between RA and ILD, but there are minority racial differences. Based on our findings, it is reasonable to consider promoting a routine screening of ILD in RA patients. Proper management of inflammation of RA is essential for downregulating the risk of ILD. This result may facilitate future insights into the biological relationship between RA and ILD.

## Data Availability

The original contributions presented in the study are included in the article/Supplementary Material; further inquiries can be directed to the corresponding author.
