# Supplementary material for: Genetic evidence reveals a causal relationship between rheumatoid arthritis and interstitial lung disease
Source: Front Genet. 2024 May 14;15:1395315. doi: 10.3389/fgene.2024.1395315 (PMC11130360; doi:10.3389/fgene.2024.1395315)
Supplement: Supplementary file 2 [file Table2.DOCX]

Supplementary table 2: Summary of the 84 SNPs in forward MR from the group of European.

|  |  |  |  |  |  |  | RA(exposure) | | | ILD(outcome) | | |
| --- | --- | --- | --- | --- | --- | --- | --- | --- | --- | --- | --- | --- |
| IVs | SNP | Chr | Position | Effect allele | Other allele | F-statistic | Beta | SE | P value | Beta | SE | P value |
| 1 | rs10175798 | 2 | 30449594 | A | G | 27.86622231 | 0.0861777 | 0.0163251 | 1.30E-07 | -0.0084 | 0.0331 | 0.8005 |
| 2 | rs1042153 | 6 | 33048663 | A | G | 314.4038429 | -0.371064 | 0.0209269 | 2.40E-70 | -0.0962 | 0.0416 | 0.02071 |
| 3 | rs10443 | 14 | 69260290 | T | C | 24.61501826 | 0.0861777 | 0.0173698 | 7.00E-07 | -0.0294 | 0.0357 | 0.4101 |
| 4 | rs10790268 | 11 | 118729391 | G | A | 62.07897928 | 0.162519 | 0.0206268 | 3.30E-15 | 0.0579 | 0.0434 | 0.1827 |
| 5 | rs10798056 | 1 | 186711910 | G | A | 21.08224371 | 0.0943107 | 0.0205401 | 4.40E-06 | -0.0634 | 0.0392 | 0.1062 |
| 6 | rs10842364 | 12 | 24678572 | A | G | 20.36472717 | 0.0953102 | 0.0211203 | 6.40E-06 | -0.0434 | 0.0329 | 0.1873 |
| 7 | rs10985070 | 9 | 123636121 | A | C | 31.81033859 | -0.0833816 | 0.0147838 | 1.70E-08 | -0.0951 | 0.033 | 0.00394003 |
| 8 | rs11075010 | 16 | 11826013 | T | C | 25.79336544 | 0.0861777 | 0.0169684 | 3.80E-07 | 0.0454 | 0.0332 | 0.1717 |
| 9 | rs11089637 | 22 | 21979096 | C | T | 25.04538207 | 0.105361 | 0.0210531 | 5.60E-07 | -0.0176 | 0.0374 | 0.637699 |
| 10 | rs11102660 | 1 | 114150717 | G | A | 23.28064698 | -0.0953102 | 0.0197534 | 1.40E-06 | -0.043 | 0.0434 | 0.3217 |
| 11 | rs11574914 | 9 | 34710338 | A | G | 54.57048351 | 0.122218 | 0.0165446 | 1.50E-13 | -0.009 | 0.0368 | 0.8073 |
| 12 | rs12126142 | 1 | 154425456 | A | G | 21.03910753 | -0.0725707 | 0.0158215 | 4.50E-06 | -0.0878 | 0.0359 | 0.0145399 |
| 13 | rs12137270 | 1 | 117264336 | T | C | 28.75823095 | 0.0953102 | 0.0177729 | 8.20E-08 | 0.0018 | 0.0381 | 0.9615 |
| 14 | rs12232497 | 17 | 38040119 | C | T | 34.82901639 | 0.0943107 | 0.0159805 | 3.60E-09 | 0.0341 | 0.0329 | 0.2997 |
| 15 | rs1234313 | 1 | 173166247 | G | A | 19.64995663 | 0.0725707 | 0.0163712 | 9.30E-06 | 0.0425 | 0.0353 | 0.2283 |
| 16 | rs12539741 | 7 | 128596805 | T | C | 45.97130691 | 0.157004 | 0.0231562 | 1.20E-11 | 0.1381 | 0.0458 | 0.00256301 |
| 17 | rs12764378 | 10 | 63800004 | A | G | 54.10525646 | 0.131028 | 0.0178133 | 1.90E-13 | -0.043 | 0.0419 | 0.3046 |
| 18 | rs13196363 | 6 | 31098391 | T | C | 104.1242576 | -0.274437 | 0.0268947 | 1.90E-24 | 0.0304 | 0.0479 | 0.5253 |
| 19 | rs13426947 | 2 | 191933254 | A | G | 49.1263451 | 0.131028 | 0.0186942 | 2.40E-12 | 0.0834 | 0.0378 | 0.0272603 |
| 20 | rs1516971 | 8 | 129542100 | C | T | 25.15164619 | -0.122218 | 0.0243698 | 5.30E-07 | 0.0163 | 0.051 | 0.749 |
| 21 | rs1571878 | 6 | 167540842 | T | C | 61.30077778 | -0.116534 | 0.014884 | 4.90E-15 | -0.0563 | 0.0332 | 0.0894108 |
| 22 | rs1633360 | 12 | 58108052 | T | C | 28.55644691 | 0.0861777 | 0.0161266 | 9.10E-08 | 0.0203 | 0.033 | 0.538801 |
| 23 | rs17264332 | 6 | 138005515 | G | A | 78.73552445 | 0.162519 | 0.0183155 | 7.10E-19 | -0.0131 | 0.042 | 0.755 |
| 24 | rs1950897 | 14 | 68760141 | T | C | 26.6020357 | 0.0861777 | 0.0167085 | 2.50E-07 | 0.0276 | 0.0355 | 0.4365 |
| 25 | rs2069235 | 22 | 39747780 | A | G | 39.67457119 | 0.10436 | 0.0165683 | 3.00E-10 | -0.0785 | 0.0349 | 0.0245901 |
| 26 | rs212389 | 6 | 159489791 | A | G | 37.13924177 | 0.0953102 | 0.0156395 | 1.10E-09 | -0.0479 | 0.0363 | 0.1874 |
| 27 | rs2233434 | 6 | 44232920 | G | A | 30.52262393 | 0.287682 | 0.0520717 | 3.30E-08 | 0.0313 | 0.0657 | 0.633501 |
| 28 | rs2240336 | 1 | 17674402 | T | C | 36.66918076 | -0.105361 | 0.0173992 | 1.40E-09 | 0.0136 | 0.0335 | 0.683701 |
| 29 | rs2253125 | 3 | 128329793 | C | T | 27.13220835 | 0.174353 | 0.0334724 | 1.90E-07 | -0.0248 | 0.0614 | 0.6859 |
| 30 | rs2343306 | 10 | 82230146 | T | C | 19.96185091 | -0.0833816 | 0.0186625 | 7.90E-06 | -0.0477 | 0.0376 | 0.2049 |
| 31 | rs244689 | 5 | 133422816 | G | A | 20.48820832 | -0.10436 | 0.0230559 | 6.00E-06 | -0.033 | 0.0378 | 0.3829 |
| 32 | rs2476601 | 1 | 114377568 | G | A | 678.2551357 | -0.593327 | 0.0227823 | 1.60E-149 | -0.0544 | 0.0463 | 0.2398 |
| 33 | rs2561477 | 5 | 102608924 | A | G | 38.60115167 | -0.105361 | 0.0169582 | 5.20E-10 | -0.0371 | 0.0354 | 0.2944 |
| 34 | rs2664035 | 4 | 48220839 | A | G | 22.16650179 | 0.076961 | 0.0163464 | 2.50E-06 | -0.0357 | 0.0345 | 0.3001 |
| 35 | rs267949 | 5 | 10743929 | C | T | 24.06794977 | -0.0861777 | 0.0175661 | 9.30E-07 | -0.022 | 0.0398 | 0.5811 |
| 36 | rs28411352 | 1 | 38278579 | T | C | 34.11309427 | 0.10436 | 0.0178679 | 5.20E-09 | -0.0226 | 0.0361 | 0.531401 |
| 37 | rs2844456 | 6 | 31864674 | C | T | 932.9046329 | 0.891598 | 0.0291911 | 1.00E-200 | -0.0213 | 0.0894 | 0.8115 |
| 38 | rs28500514 | 8 | 130609068 | C | T | 19.53071455 | 0.0725707 | 0.0164211 | 9.90E-06 | 0.0068 | 0.0332 | 0.8374 |
| 39 | rs3087243 | 2 | 204738919 | A | G | 82.77339454 | -0.139262 | 0.0153069 | 9.20E-20 | -0.0153 | 0.0348 | 0.6609 |
| 40 | rs3130761 | 6 | 29073688 | T | C | 27.72281417 | 0.24686 | 0.0468848 | 1.40E-07 | 0.098 | 0.0937 | 0.2953 |
| 41 | rs34046593 | 4 | 26111593 | A | G | 69.13361899 | 0.139762 | 0.0168091 | 9.20E-17 | 0.0509 | 0.0353 | 0.1498 |
| 42 | rs35515539 | 4 | 66354218 | A | G | 22.01884155 | -0.116534 | 0.0248345 | 2.70E-06 | 0.0031 | 0.0443 | 0.9443 |
| 43 | rs41264261 | 1 | 203743798 | T | C | 20.52035629 | -0.328504 | 0.0725184 | 5.90E-06 | -0.0042 | 0.0893 | 0.9625 |
| 44 | rs4239702 | 20 | 44749251 | C | T | 57.07239393 | 0.139262 | 0.018434 | 4.20E-14 | 0.0613 | 0.0362 | 0.0905191 |
| 45 | rs4566053 | 13 | 42999179 | T | C | 21.46689213 | -0.0833816 | 0.0179964 | 3.60E-06 | 0.0133 | 0.0429 | 0.755899 |
| 46 | rs4657041 | 1 | 161478859 | C | T | 28.41320183 | -0.0861777 | 0.0161672 | 9.80E-08 | -3.00E-04 | 0.0328 | 0.9931 |
| 47 | rs4771205 | 13 | 28618314 | A | G | 20.33497188 | -0.0943107 | 0.0209141 | 6.50E-06 | -0.029 | 0.0393 | 0.4612 |
| 48 | rs4958880 | 5 | 150438477 | A | C | 24.3338356 | 0.0953102 | 0.0193212 | 8.10E-07 | -0.012 | 0.0428 | 0.778599 |
| 49 | rs507201 | 8 | 102462292 | C | T | 27.72271195 | -0.0861777 | 0.0163673 | 1.40E-07 | -0.0223 | 0.0406 | 0.582599 |
| 50 | rs537544 | 10 | 8108382 | T | C | 42.25804987 | -0.116534 | 0.0179266 | 8.00E-11 | 0.0857 | 0.0335 | 0.0105201 |
| 51 | rs56269853 | 10 | 6517619 | T | C | 21.81655408 | -0.0833816 | 0.0178516 | 3.00E-06 | -0.0035 | 0.0349 | 0.9199 |
| 52 | rs56656810 | 16 | 30788759 | A | C | 21.94897859 | 0.076961 | 0.0164272 | 2.80E-06 | -0.006 | 0.0378 | 0.873 |
| 53 | rs57002461 | 5 | 84470583 | G | A | 20.91545085 | 0.198451 | 0.043393 | 4.80E-06 | 0.0775 | 0.0865 | 0.37 |
| 54 | rs57585717 | 7 | 28149255 | A | G | 27.4643784 | -0.127833 | 0.0243926 | 1.60E-07 | -0.1095 | 0.0564 | 0.05223 |
| 55 | rs592390 | 18 | 12822314 | C | T | 34.7234128 | -0.0953102 | 0.0161744 | 3.80E-09 | -0.0113 | 0.0328 | 0.7317 |
| 56 | rs60733400 | 1 | 2516781 | A | G | 36.81376135 | -0.105361 | 0.017365 | 1.30E-09 | 0.0069 | 0.0342 | 0.8391 |
| 57 | rs61511343 | 3 | 156831730 | A | G | 22.41194645 | 0.285179 | 0.060239 | 2.20E-06 | -0.1218 | 0.0804 | 0.1298 |
| 58 | rs61828284 | 1 | 173299743 | T | C | 33.11201994 | -0.198451 | 0.0344874 | 8.70E-09 | -0.0585 | 0.0665 | 0.379 |
| 59 | rs62321692 | 4 | 123261530 | C | A | 21.69262061 | 0.127833 | 0.0274465 | 3.20E-06 | 0.0846 | 0.0587 | 0.1499 |
| 60 | rs6442323 | 3 | 12722870 | G | A | 20.27709681 | -0.0861777 | 0.0191378 | 6.70E-06 | 0.0363 | 0.033 | 0.2714 |
| 61 | rs6679356 | 1 | 67820194 | T | C | 21.03920116 | -0.0943107 | 0.0205611 | 4.50E-06 | 0.1282 | 0.0558 | 0.0216202 |
| 62 | rs671703 | 6 | 138147365 | G | A | 20.87609062 | -0.113329 | 0.0248037 | 4.90E-06 | -0.0121 | 0.0474 | 0.7988 |
| 63 | rs6930468 | 6 | 426268 | G | A | 27.46456932 | 0.0833816 | 0.0159105 | 1.60E-07 | 0.0252 | 0.034 | 0.4593 |
| 64 | rs706778 | 10 | 6098949 | T | C | 46.99981791 | 0.10436 | 0.0152225 | 7.10E-12 | 0.015 | 0.0329 | 0.648 |
| 65 | rs7224929 | 17 | 5011736 | A | G | 25.34250801 | 0.173953 | 0.0345547 | 4.80E-07 | -0.0821 | 0.052 | 0.1147 |
| 66 | rs7277167 | 21 | 34773777 | T | C | 25.95197443 | -0.127833 | 0.0250933 | 3.50E-07 | 0.0209 | 0.0472 | 0.6584 |
| 67 | rs73000522 | 11 | 107966040 | T | C | 25.46718934 | -0.18633 | 0.0369226 | 4.50E-07 | -0.0587 | 0.0474 | 0.2152 |
| 68 | rs73081554 | 3 | 58302935 | T | C | 29.83663305 | 0.165514 | 0.0303012 | 4.70E-08 | 0.0214 | 0.0694 | 0.7582 |
| 69 | rs73371668 | 6 | 15145022 | G | A | 20.91553428 | 0.116534 | 0.0254811 | 4.80E-06 | -0.1208 | 0.0656 | 0.0656599 |
| 70 | rs7660626 | 4 | 10727372 | A | G | 24.94480545 | -0.105361 | 0.0210955 | 5.90E-07 | -0.035 | 0.0335 | 0.2963 |
| 71 | rs7731626 | 5 | 55444683 | A | G | 96.74100948 | -0.198451 | 0.0201766 | 7.90E-23 | -0.0739 | 0.0366 | 0.0437703 |
| 72 | rs7927748 | 11 | 128499574 | T | G | 24.94454481 | 0.076961 | 0.0154093 | 5.90E-07 | -0.0037 | 0.0332 | 0.9106 |
| 73 | rs8026898 | 15 | 69991417 | A | G | 71.78450094 | 0.14842 | 0.0175177 | 2.40E-17 | -0.0399 | 0.0364 | 0.2725 |
| 74 | rs8032939 | 15 | 38834033 | C | T | 49.12630299 | 0.116534 | 0.0166263 | 2.40E-12 | -0.0384 | 0.0365 | 0.2929 |
| 75 | rs8133843 | 21 | 36738242 | A | G | 33.83488199 | 0.0953102 | 0.0163854 | 6.00E-09 | 0.0317 | 0.0343 | 0.3547 |
| 76 | rs867768 | 10 | 31414304 | T | C | 21.75362218 | 0.0861777 | 0.0184769 | 3.10E-06 | 0.0298 | 0.0347 | 0.3901 |
| 77 | rs9261602 | 2 | 76934183 | G | A | 75.08886449 | 0.18633 | 0.0215028 | 4.50E-18 | 0.0236 | 0.0328 | 0.473 |
| 78 | rs9310852 | 3 | 27784997 | G | A | 30.58230767 | 0.0833816 | 0.0150777 | 3.20E-08 | 0.0394 | 0.0328 | 0.2297 |
| 79 | rs9368695 | 6 | 31453781 | T | C | 31.92798081 | -0.356675 | 0.0631229 | 1.60E-08 | 0.0115 | 0.1184 | 0.9228 |
| 80 | rs9603608 | 13 | 40318819 | C | A | 42.35801786 | -0.10436 | 0.0160349 | 7.60E-11 | -0.0511 | 0.0342 | 0.1344 |
| 81 | rs9653442 | 2 | 100825367 | T | C | 48.33154658 | -0.105361 | 0.0151553 | 3.60E-12 | -0.0108 | 0.033 | 0.744699 |
| 82 | rs968567 | 11 | 61595564 | T | C | 24.50837313 | -0.105361 | 0.0212825 | 7.40E-07 | -0.0112 | 0.0529 | 0.8327 |
| 83 | rs9826828 | 3 | 136402060 | A | G | 28.53541642 | 0.350657 | 0.0656433 | 9.20E-08 | 0.1104 | 0.3904 | 0.777401 |
| 84 | rs998731 | 8 | 81095395 | T | C | 27.86622231 | 0.0861777 | 0.0163251 | 1.30E-07 | 0.0156 | 0.033 | 0.6363 |

RA, Rheumatoid arthritis; ILD, Interstitial lung disease; SNP, single nucleotide polymorphism; MR: Mendelian randomization; Chr, chromosome; SE, standard error; Beta, effect size (log(OR) scale) estimated with revenue for the alternative allele.
