# Supplementary material for: Genetic evidence reveals a causal relationship between rheumatoid arthritis and interstitial lung disease
Source: Front Genet. 2024 May 14;15:1395315. doi: 10.3389/fgene.2024.1395315 (PMC11130360; doi:10.3389/fgene.2024.1395315)
Supplement: Supplementary file 3 [file Table5.DOCX]

Supplementary table 5: Summary of the 12 SNPs in reverse MR from the group of East Asian.

|  |  |  |  |  |  |  | ILD(exposure) | | | RA(outcome) | | |
| --- | --- | --- | --- | --- | --- | --- | --- | --- | --- | --- | --- | --- |
| IVs | SNP | Chr | Position | Effect allele | Other allele | F-statistic | Beta | SE | P value | Beta | SE | P value |
| 1 | rs10063294 | 5 | 35877505 | A | G | 26.26077653 | -0.244422 | 0.0476965 | 2.98E-07 | 0.0016639 | 0.0218002 | 0.93916 |
| 2 | rs10416053 | 19 | 13460800 | C | T | 22.29110396 | 0.309674 | 0.0655902 | 2.34E-06 | 0.0232562 | 0.0296834 | 0.433348 |
| 3 | rs11754421 | 6 | 7563564 | T | A | 20.17689041 | 0.198555 | 0.0442032 | 7.06E-06 | -0.00377674 | 0.0202274 | 0.851885 |
| 4 | rs143714555 | 9 | 90549412 | G | C | 24.58551627 | 0.314086 | 0.0633445 | 7.11E-07 | -0.00805237 | 0.028807 | 0.779839 |
| 5 | rs56167726 | 7 | 128560902 | T | C | 19.69485633 | -0.206996 | 0.0466429 | 9.09E-06 | 0.00670585 | 0.0213 | 0.752891 |
| 6 | rs56345976 | 5 | 1276873 | A | G | 20.86024897 | -0.249799 | 0.0546929 | 4.94E-06 | 0.00750799 | 0.025091 | 0.764765 |
| 7 | rs6071504 | 20 | 59702457 | T | C | 19.65153338 | -0.381965 | 0.0861639 | 9.29E-06 | -0.0506014 | 0.0386274 | 0.190199 |
| 8 | rs6477542 | 9 | 109507432 | T | C | 29.63650303 | 0.241674 | 0.0443932 | 5.21E-08 | -0.0213988 | 0.0202997 | 0.291819 |
| 9 | rs6586981 | 8 | 21210074 | T | C | 21.14115054 | 0.271303 | 0.0590052 | 4.27E-06 | 0.0257498 | 0.0268317 | 0.337217 |
| 10 | rs75420072 | 5 | 156326773 | A | T | 22.10998341 | 0.623657 | 0.132633 | 2.57E-06 | 0.0126445 | 0.0574182 | 0.825702 |
| 11 | rs7734992 | 5 | 1280128 | C | T | 39.06358301 | -0.315605 | 0.0504961 | 4.10E-10 | -0.00529933 | 0.0230482 | 0.818151 |
| 12 | rs9653635 | 20 | 19924182 | T | C | 20.71462346 | 0.273069 | 0.0599976 | 5.33053E-06 | 0.031187 | 0.0273217 | 0.253674 |

RA, Rheumatoid arthritis; ILD, Interstitial lung disease; SNP, single nucleotide polymorphism; MR: Mendelian randomization; Chr, chromosome; SE, standard error; Beta, effect size (log(OR) scale) estimated with revenue for the alternative allele.
