# Supplementary material for: Genetic evidence reveals a causal relationship between rheumatoid arthritis and interstitial lung disease
Source: Front Genet. 2024 May 14;15:1395315. doi: 10.3389/fgene.2024.1395315 (PMC11130360; doi:10.3389/fgene.2024.1395315)
Supplement: Supplementary file 4 [file Table3.DOCX]

Supplementary table 3: Summary of the 8 SNPs in reverse MR from the group of European.

|  |  |  |  |  |  |  | ILD(exposure) | | | RA(outcome) | | |
| --- | --- | --- | --- | --- | --- | --- | --- | --- | --- | --- | --- | --- |
| IVs | SNP | Chr | Position | Effect allele | Other allele | F-statistic | Beta | SE | P value | Beta | SE | P value |
| 1 | rs10811936 | 9 | 23602407 | A | C | 19.63295537 | 0.2437 | 0.055 | 9.46E-06 | -0.0100503 | 0.0497804 | 0.84 |
| 2 | rs10973140 | 9 | 36957447 | T | C | 21.23551144 | 0.2023 | 0.0439 | 4.12E-06 | 0.0487902 | 0.0285984 | 0.0879995 |
| 3 | rs11617392 | 13 | 47279258 | T | G | 20.22128678 | 0.4227 | 0.094 | 6.94E-06 | -0.0833816 | 0.0564996 | 0.14 |
| 4 | rs2076295 | 6 | 7563232 | G | T | 19.65706007 | 0.1503 | 0.0339 | 9.18E-06 | 0.0304592 | 0.020117 | 0.13 |
| 5 | rs2141595 | 3 | 169503432 | T | C | 19.67469319 | 0.1619 | 0.0365 | 9.24E-06 | 0.0392207 | 0.0156124 | 0.012 |
| 6 | rs7734992 | 5 | 1280128 | C | T | 26.69444444 | -0.1705 | 0.033 | 2.40E-07 | -0.0582689 | 0.0384841 | 0.13 |
| 7 | rs9317562 | 13 | 66659373 | G | T | 23.37031837 | -0.1692 | 0.035 | 1.36E-06 | -0.0198026 | 0.0187149 | 0.29 |
| 8 | rs9809990 | 3 | 168871394 | G | A | 30.96437879 | 0.1853 | 0.0333 | 2.72E-08 | 0.0100503 | 0.0191654 | 0.6 |

RA, Rheumatoid arthritis; ILD, Interstitial lung disease; SNP, single nucleotide polymorphism; MR: Mendelian randomization; Chr, chromosome; SE, standard error; Beta, effect size (log(OR) scale) estimated with revenue for the alternative allele.
