# Supplementary material for: Genetic evidence reveals a causal relationship between rheumatoid arthritis and interstitial lung disease
Source: Front Genet. 2024 May 14;15:1395315. doi: 10.3389/fgene.2024.1395315 (PMC11130360; doi:10.3389/fgene.2024.1395315)
Supplement: Supplementary file 5 [file Table4.DOCX]

Supplementary table 4: Summary of the 36 SNPs in forward MR from the group of East Asian.

|  |  |  |  |  |  |  | RA(exposure) | | | ILD(outcome) | | |
| --- | --- | --- | --- | --- | --- | --- | --- | --- | --- | --- | --- | --- |
| IVs | SNP | Chr | Position | Effect allele | Other allele | F-statistic | Beta | SE | P value | Beta | SE | P value |
| 1 | rs11889341 | 2 | 191943742 | T | C | 33.6377446 | 0.127072 | 0.0219097 | 6.64E-09 | 0.129349 | 0.0478322 | 0.00684621 |
| 2 | rs11947354 | 4 | 183723017 | A | G | 19.56319675 | 0.136734 | 0.0309141 | 9.73E-06 | -0.0186965 | 0.0674604 | 0.781666 |
| 3 | rs12439845 | 15 | 41211827 | C | T | 24.96778044 | 0.110075 | 0.0220292 | 5.83E-07 | 0.0491291 | 0.0480812 | 0.306878 |
| 4 | rs12805524 | 11 | 128395947 | G | A | 21.69398026 | -0.123694 | 0.026557 | 3.20E-06 | -0.0877795 | 0.057836 | 0.129082 |
| 5 | rs13001834 | 2 | 233824870 | C | A | 20.10041156 | 0.133719 | 0.0298257 | 7.35E-06 | 0.0937218 | 0.0649011 | 0.148719 |
| 6 | rs1362076 | 6 | 29441169 | T | G | 72.95169238 | -0.330207 | 0.0386606 | 1.33E-17 | 0.0267284 | 0.0827034 | 0.746555 |
| 7 | rs199949106 | 6 | 30692754 | T | C | 21.63025534 | -0.126585 | 0.0272177 | 3.31E-06 | -0.0109198 | 0.0596617 | 0.854775 |
| 8 | rs2203196 | 10 | 5908209 | A | G | 19.86011103 | 0.175432 | 0.0393657 | 8.33E-06 | 0.0416403 | 0.0861455 | 0.628831 |
| 9 | rs2235369 | 6 | 14118505 | A | G | 24.25047965 | 0.139279 | 0.028283 | 8.46E-07 | -0.101139 | 0.0617447 | 0.101418 |
| 10 | rs2256726 | 10 | 31286821 | C | T | 20.80832354 | 0.0942653 | 0.0206649 | 5.08E-06 | 0.0378764 | 0.045035 | 0.400323 |
| 11 | rs2294360 | 22 | 39897792 | A | G | 19.92350635 | -0.137034 | 0.0307005 | 8.06E-06 | -0.119372 | 0.0669811 | 0.0747205 |
| 12 | rs28362859 | 6 | 44228815 | C | A | 47.98048777 | 0.172117 | 0.024848 | 4.31E-12 | 0.0453857 | 0.0540023 | 0.400662 |
| 13 | rs2841280 | 14 | 105393556 | C | G | 23.58539506 | 0.102107 | 0.0210249 | 1.19E-06 | 0.113119 | 0.0459147 | 0.0137521 |
| 14 | rs34493748 | 6 | 33001771 | C | T | 61.03463175 | -0.385471 | 0.0493405 | 5.61E-15 | -0.0585665 | 0.104202 | 0.574082 |
| 15 | rs3910172 | 10 | 64067508 | T | C | 27.52485792 | -0.175481 | 0.0334478 | 1.55E-07 | -0.125622 | 0.0723963 | 0.0827066 |
| 16 | rs4350841 | 21 | 45723628 | C | T | 27.95914484 | 0.109828 | 0.0207707 | 1.24E-07 | -7.33E-05 | 0.045435 | 0.998712 |
| 17 | rs4548024 | 6 | 138165744 | C | T | 20.15070926 | 0.147683 | 0.0328992 | 7.16E-06 | 0.207548 | 0.0725642 | 0.00423389 |
| 18 | rs4944668 | 11 | 72401101 | C | T | 20.24712626 | 0.116256 | 0.0258365 | 6.81E-06 | 0.0795958 | 0.056622 | 0.159801 |
| 19 | rs542715 | 8 | 27551073 | T | C | 20.09917074 | 0.0904779 | 0.0201815 | 7.35E-06 | 0.1098 | 0.0440197 | 0.0126194 |
| 20 | rs564419538 | 6 | 32008417 | C | G | 109.9538134 | 0.280617 | 0.0267614 | 1.00E-25 | 0.0343864 | 0.0574825 | 0.549703 |
| 21 | rs58107865 | 4 | 109061618 | C | G | 30.04919793 | -0.14336 | 0.0261524 | 4.21E-08 | -0.00686878 | 0.0570123 | 0.904104 |
| 22 | rs6461145 | 7 | 15026872 | A | G | 24.52112047 | 0.100644 | 0.0203244 | 7.35E-07 | 0.0339873 | 0.0443928 | 0.443912 |
| 23 | rs6570194 | 6 | 138242638 | C | A | 27.57836821 | 0.207483 | 0.0395092 | 1.51E-07 | 0.262511 | 0.0868718 | 0.00251264 |
| 24 | rs657555 | 18 | 12847136 | T | C | 20.11996061 | -0.0945482 | 0.0210785 | 7.27E-06 | -0.0668608 | 0.0461396 | 0.147311 |
| 25 | rs67140765 | 6 | 31380787 | T | G | 146.2976781 | 0.390039 | 0.032247 | 1.12E-33 | 0.249464 | 0.0681376 | 0.000251061 |
| 26 | rs7172228 | 15 | 53059683 | T | G | 22.35982185 | 0.224432 | 0.0474625 | 2.26E-06 | 0.0654466 | 0.102683 | 0.523886 |
| 27 | rs72895826 | 1 | 54874777 | G | C | 20.5868449 | -0.205239 | 0.045234 | 5.70E-06 | -0.185663 | 0.0987559 | 0.0601063 |
| 28 | rs741242 | 12 | 4249427 | C | A | 21.45836261 | 0.202971 | 0.0438163 | 3.62E-06 | 0.00796896 | 0.0938449 | 0.932328 |
| 29 | rs76153210 | 6 | 44284508 | T | C | 30.74280219 | 0.137338 | 0.0247696 | 2.95E-08 | 0.0209636 | 0.0539015 | 0.697333 |
| 30 | rs76895136 | 5 | 79577762 | G | A | 21.89648279 | 0.180625 | 0.0386003 | 2.88E-06 | 0.00814004 | 0.0846433 | 0.923386 |
| 31 | rs7731626 | 5 | 55444683 | A | G | 20.724594 | -0.164766 | 0.036193 | 5.30E-06 | 0.0983209 | 0.0779253 | 0.207045 |
| 32 | rs78897192 | 1 | 161595080 | T | A | 25.46287431 | -0.128027 | 0.0253716 | 4.51E-07 | -0.00674626 | 0.0553774 | 0.903039 |
| 33 | rs7990 | 6 | 32609965 | A | C | 646.3561501 | 0.583582 | 0.0229544 | 1.39E-142 | 0.141347 | 0.048531 | 0.00358525 |
| 34 | rs8068939 | 17 | 78275265 | A | C | 20.18584544 | -0.174892 | 0.0389266 | 7.03E-06 | -0.0158145 | 0.0839755 | 0.850624 |
| 35 | rs9258276 | 6 | 29731791 | T | C | 62.33977023 | -0.208036 | 0.0263485 | 2.89E-15 | 0.0411814 | 0.057567 | 0.474384 |
| 36 | rs9269271 | 6 | 32539107 | C | T | 159.501593 | -0.365674 | 0.0289542 | 1.45445E-36 | -0.0239884 | 0.061726 | 0.697553 |

RA, Rheumatoid arthritis; ILD, Interstitial lung disease; SNP, single nucleotide polymorphism; MR: Mendelian randomization; Chr, chromosome; SE, standard error; Beta, effect size (log(OR) scale) estimated with revenue for the alternative allele.
